# Cross-sectional associations between the neighborhood built environment and physical activity in a rural setting: the Bogalusa Heart Study

**DOI:** 10.1186/s12889-020-09509-4

**Published:** 2020-09-18

**Authors:** Jeanette Gustat, Christopher E. Anderson, Queendaleen C. Chukwurah, Maeve E. Wallace, Stephanie T. Broyles, Lydia A. Bazzano

**Affiliations:** 1 Department of Epidemiology, School of Public Health and Tropical Medicine, Tulane University, 1440 Canal Street, Suite 2001, New Orleans, LA 70112 USA; 2 Center for Aging, Tulane University School of Medicine, New Orleans, LA 70112 USA; 3 Department of Global Community Health and Behavioral Sciences, School of Public Health and Tropical Medicine, Tulane University, New Orleans, LA 70112 USA; 4 Pennington Biomedical Research Center, Louisiana State University, Baton Rouge, LA 70808 USA

**Keywords:** Physical activity, Built environment, Rural

## Abstract

**Background:**

Insufficient physical activity (PA) is a common health risk and more prevalent in rural populations. Few studies have assessed relationships between the built environment and PA in rural settings, and community policy guidance to promote PA through built environment interventions is primarily based on evidence from urban studies.

**Methods:**

Participants in the Bogalusa Heart Study, a longitudinal study in rural Louisiana, with International Physical Activity Questionnaire data from 2012 to 2013 and a valid residential address (*N* = 1245) were included. PA was summarized as the number of weekly metabolic equivalent (MET)-minutes of total, transportation, and leisure time PA. The Rural Active Living Assessment street segment audit tool and Google Street View were used to assess features of the built environment overall and in six categories (path features, pedestrian safety features, aesthetics, physical security, destinations and land use) that influence PA. Scores for street segment built environment (overall and in categories) were calculated, for segments and buffers of 0.25, 0.50, 1.00 and 1.50 miles. Associations between built environment scores and PA were assessed with generalized estimating equations.

**Results:**

Participants reported little weekly total, leisure time, and transportation PA (mean 470, 230 and 43 MET-minutes per week, respectively). A 1-point increase in the overall built environment score was associated with 10.30 additional weekly leisure time MET-minutes within a 1.50 mile buffer (*p*-value 0.05), with a similar magnitude observed for a 1.00-mile buffer. A 1-point increase in the aesthetic score was associated with significantly higher leisure time PA for all geographic units (from 22.21 to 38.75 MET-minutes weekly) when adjusted for individual covariates, but was attenuated and only significant for the segment of the residence after accounting for other neighborhood characteristics.

**Conclusions:**

Significant associations between features of the environment (overall and aesthetic scores) with leisure time PA were observed among adults in this rural population. Built environment interventions in rural settings face additional barriers of lower population density and greater distances for infrastructure projects, and it is important to identify approaches that are both feasible for rural communities and can promote PA.

## Introduction

Physical inactivity is a prevalent behavior associated with increased risk of many chronic health conditions and a detriment to public health [1]. Physical inactivity is a leading cause of mortality [2], and contributes substantially to healthcare costs [3]. Adults in the United States are recommended to get either ≥75 min of vigorous or ≥ 150 min of moderate-to-vigorous physical activity (PA) per week [4]. Nearly 32% of men and half of women in the United States report receiving insufficient PA [5].

Rural populations in the United States are recognized as a health disparity population due to the elevated prevalence of disease and early mortality, both of which are substantially higher in rural populations than among the general adult population of the United States population [6]. There is a need to improve behaviors to reduce disease risk in rural populations [7]. Behaviors that contribute to elevated health risk, including smoking, being overweight or obese, and failure to meet PA guidelines are all more prevalent among rural populations in the United States [8, 9]. Over 69% of adults 18 years of age or older living in micropolitan and noncore (i.e. non-metropolitan and rural) counties reported not meeting guidelines for aerobic PA, which was significantly higher than in any metropolitan county classification (i.e. counties including small or large urban areas) [9].

The amount of PA an individual will engage in is a product of the characteristics of individuals and environmental features that promote or deter PA when individuals interact with them [10]. Research into urban-rural disparities in PA has identified lower accessibility of PA facilities and resources among rural residents [11]. Built environment (BE) features differ between rural and urban locales, with additional impediments to PA and active transportation including greater distances to travel, roads with higher speed limits, and fewer sidewalks, crosswalks and bike lanes in rural areas [12]. Within rural areas, as density decreases the availability of sidewalks decreases and land use mixture for PA deteriorates, indicating that as population density declines in rural areas, the BE becomes less conducive to PA [13]. Relatedly, as degree of urbanization increases, opportunities to engage in PA increase [14]. Associations between BE and PA have been identified, with a systematic review of the literature reporting that pleasant aesthetics, the presence of trails, parks and walkable destinations and lower levels of crime were associated with more PA in adults who live in rural areas [15]. Understanding the physical and contextual challenges of rural communities is important to promoting PA in rural settings [16, 17]. Promoting PA in rural communities has unique challenges that relate not only to cultural differences, population size and policy differences but also environment features [16]. However, research into the relationship between the BE and PA in rural populations has been limited. It is important to assess BE features in relation to domains of PA (i.e. total, leisure time, transport) so that the relationships between the BE features and PA in specific domains are not obscured [18]. Additionally, no studies have linked the BE in variable-sized geographic units to individual resident data in a rural setting. This study was conducted to develop summary scores for rural BE and evaluate the relationship between BE features around the residence and total, leisure time and transport PA in a population of adults in a small community in the rural southern United States.

## Methods

The Bogalusa Heart Study (BHS) is a long-running study of cardiovascular risk factors and disease from childhood into adulthood conducted in a rural area (Washington Parish, Louisiana) of the southern United States. The population of the largest town in the parish, Bogalusa, is currently 11,706. It was between 12,030 and 11,920 in 2012–2013. This study included current participants in the most recent BHS study visits, who had complete PA data the last time it was assessed in 2012–2013 and a valid address of residence to allow the auditing of the BE around the residence (*n* = 1245) and the assessment of cross-sectional associations between BE around the residence and PA.

Street segments of the participants in this study were audited for BE features as part of a larger BE assessment for the Bogalusa Heart Study. The Rural Active Living Assessment (RALA) [19] street segment audit tool was used for observations of segments in this study. A total of 1340 street segments of residence were identified among Bogalusa Heart Study participants, and 2648 audits of these segments were conducted using Google StreetView. Because Google StreetView caches images from multiple years, audits were conducted of all available images for each segment (generally 2008 and 2014 for segments in Washington Parish, Louisiana). Duplicate assessment of specific street segments and image years were conducted for 196 randomly selected segments (14.6% of all segments), and agreement on individual features and in categories of features was evaluated with percent agreement and Kappa coefficients, respectively (Supplemental Table 1 and Supplemental Table 2). Google StreetView has previously been used to assess BE features in public health research [20], and reliability of assessments using it have been reported to be high [21, 22].

PA was assessed with the International Physical Activity Questionnaire (IPAQ), with participants providing information on all items on the long form. The validity of PA assessed with IPAQ has been previously reported [23]. PA was summarized as the number of weekly metabolic equivalent (MET)-minutes for total, leisure time, and transport PA following the scoring guidance provided for IPAQ.

Study participants were characterized with anthropometric (body mass index (BMI)), demographic (age, race, sex), socioeconomic (education, income) and behavioral (smoking, alcohol consumption) variables. Age and BMI were available as continuous variables. Participant education was self-reported and categorized as greater than or equal to a high school degree or less than a high school degree. Annual self-reported income was categorized as ≥25,000 US dollars or < 25,000 US dollars. Race was self-reported as white or black. Alcohol consumption in the past 12 months was self-reported (yes or no), as was smoking (current, former, never). Additional neighborhood contextual variables were obtained for the census tract of residence from American Community Survey [24] five-year estimates (2009–2013) and the 2010 census [25]. These included the percent of residents in a census tract living in a household with an income below the federal poverty level (FPL) and population density (residents per square mile), both treated as continuous variables.

### Statistical analyses

We developed scores for street segment BE, overall and in six categories of features identified a priori and refined following principal components analysis. The categories of features identified included path features, pedestrian safety features, segment aesthetics, physical security, destinations and land use. Individual items in each category are shown in Supplemental Table 1. Briefly, the street segment BE scores are the number of additional features thought to promote PA on a street segment relative to the sample mean for each feature. A BE score of 0 indicates that the segment has no features that promote PA relative to the average street segment. This scoring process has been used in the development of walkability and playground indices [26, 27].

The scoring followed a 6-step process. First (step 1), features that were assessed across multiple variables using the RALA (i.e. the presence and characteristics of sidewalks) were combined into one variable, and all variables were coded so that higher numerical values were assigned to features thought to promote PA (i.e. the presence of a crossing signal = 1, the absence of a crossing signal = 0). Next (step 2), sample means were calculated for each variable and (step 3) 1-point was added to a preliminary score for a segment for each variable that the segment value exceeds the sample mean for that variable. This was followed (step 4) by the calculation of a mean preliminary score for segments with values above and below the sample mean for every variable. To evaluate the internal consistency of the preliminary scores, mean preliminary scores were compared for segments above and below the sample mean for each variable (step 5), and variables for which there was not a difference in mean preliminary score ≥ 1 between segments above and below the sample mean were flagged for removal. The mean preliminary scores calculated in step 4 and the variables flagged for removal in step 5 are shown in Supplemental Table 1. Finally (step 6), a final score was calculated by adding 1-point to the score for each variable that was not flagged for removal in step 5 and for which the segment value exceeds the sample mean for that variable. This scoring process was done for all variables assessed to generate an overall score, and for variables in categories of features (path, pedestrian safety, aesthetics, physical security, destinations, land use).

Because of large differences in Google StreetView image quality over time, the scoring was stratified by image year (≤ 2010 vs > 2010) and auditor. Agreement was assessed for the scores from duplicate audits with intraclass correlation coefficients and was found to be acceptable for all scores except the physical security category (Supplemental Table 3). Scores for each audit of a segment were averaged to give an average segment score. Neighborhood BE scores were then calculated as an average, weighted by the inverse distance (from the center point of each segment), of all segment scores in buffers with radii of 0.25, 0.50, 1.00 and 1.50 miles around each segment. Because this study evaluated physical activity in different domains, accommodating the potential for relevant geographic buffers for the different domains of activity was essential, with 0.25, 0.50, 1.00 and 1.50-mile buffers thought to capture built environment features relevant for short, medium and longer distance walking trips [28].

This allowed the closer segments to contribute the most to the neighborhood scores in each buffer. As an example in Supplemental Fig. 1, for street segment A, the score for a 0.25-mile buffer would include the score of street segment A, and segments 1 and 2, with segments 1 and 2 receiving lower weights than the central segment (segment A) due to their distance from the centroid. The 0.50-mile buffer score for segment A would average the scores of segments A and 1–7, with weights decreasing from segment 1 to 7 due to the increased distance from the centroid. The 1.00-mile buffer score for segment A would include all the audited segments in the figure, with segments 8, 9, 10, 11, and B contributing the least (having the smallest weights) to the weighted average score because they are the furthest from the central segment A.

The study sample was characterized with frequencies or means and standard deviations, and differences between individuals living on segments with an overall score greater or equal to versus below the average score were compared with chi-square or t-tests as appropriate. The associations between BE scores and total PA, leisure time PA and transport PA were evaluated in generalized estimating equations (GEE) linear regression models that accommodated clustering within street segments and census tracts. Minimally adjusted models were run with gender and age as covariates, and fully adjusted models additionally incorporated the percent of residents in a census tract living below the FPL and population density. *P*-values < 0.05 were considered statistically significant, and *p*-values < 0.10 were highlighted because those relationships merit further exploration.

## Results

The majority of study participants were female (59.0%), white (65.9%), and reported drinking alcohol in the last year (87.9%), never being a smoker (64.7%), having a high school degree or more education (84.3%), and having an income ≥$25,000 US dollars per year (62.3%) (Table 1). The mean age was 48.1, and mean BMI was 31.4. Participants reported low levels of PA, with only 27.0% of participants reporting enough weekly PA to meet US guidelines of 150 min of moderate to vigorous PA per week [4]. Population density at the census tract level was the only variable significantly different between individuals living on street segments above or below the sample mean for the overall score (*p* < 0.0001), with higher population density (952.0 people per square mile) observed for individuals living on street segments with higher overall scores and lower population density (330.9 residents per square mile) for individuals living on street segments with lower overall scores.

**Table 1:** Description of study participants with PA data and valid address

Variable	Full Sample	10.56 < Overall mRALA score	10.56 ≥ Overall mRALA score	
	N = 1245	*n* = 515	*n* = 730	*p*-value*
Female, n (%)	735 (59.0)	308 (59.7)	427 (58.4)	0.63
Drink alcohol, n (%)	1094 (87.9)	457 (88.9)	637 (87.5)	0.45
Smoke, n (%)				0.56
Current	245 (19.7)	94 (18.3)	151 (20.7)	
Former	195 (15.7)	83 (16.1)	112 (15.3)	
Never	805 (64.7)	338 (65.6)	467 (64.0)	
Race, n (%)				0.53
White	820 (65.9)	327 (63.6)	493 (67.5)	
Black	422 (33.9)	186 (36.2)	236 (32.3)	
Education, n (%)				0.99
< High school	196 (15.7)	81 (15.7)	115 (15.8)	
≥ High school	1049 (84.3)	434 (84.3)	615 (84.2)	
Income, n (%)				1.00
< $25,000	469 (37.7)	194 (37.7)	275 (37.7)	
≥ $25,000	776 (62.3)	321 (62.3)	455 (62.3)	
≥150 min/wk. MVPA, n (%)	336 (27.0)	144 (28.0)	192 (26.3)	0.52
Total PA (MET-minutes/wk), mean ± SD	470.3 ± 783.3	490.8 ± 788.1	455.8 ± 780.2	0.44
Transport PA (MET-minutes/wk), mean ± SD	43.5 ± 162.0	46.7 ± 160.5	41.2 ± 163.1	0.56
Leisure PA (MET-minutes/wk), mean ± SD	230.4 ± 436.6	256.1 ± 483.3	212.2 ± 399.8	0.09
BMI (kg/m^2^), mean ± SD	31.4 ± 7.8	31.3 ± 7.9	31.5 ± 7.8	0.79
Age, mean ± SD	48.1 ± 5.2	48.4 ± 5.2	47.9 ± 5.3	0.13
Tract, population density, mean ± SD	587.4 ± 1135.9	952.0 ± 1623.0	330.9 ± 432.7	< 0.0001
Tract, proportion poverty, mean ± SD	0.3 ± 0.1	0.3 ± 0.1	0.3 ± 0.1	0.45

*PA* Physical activity, *SD* standard deviation, *min* minutes, *wk.* week, *BMI* body mass index, *RALA* Rural Active Living Assessment

*p-value is for the comparison of participants living on street segments below the average overall street segment score of 10.56 to those living on a street segment at or above the mean overall street segment score

**Fig. 1:**
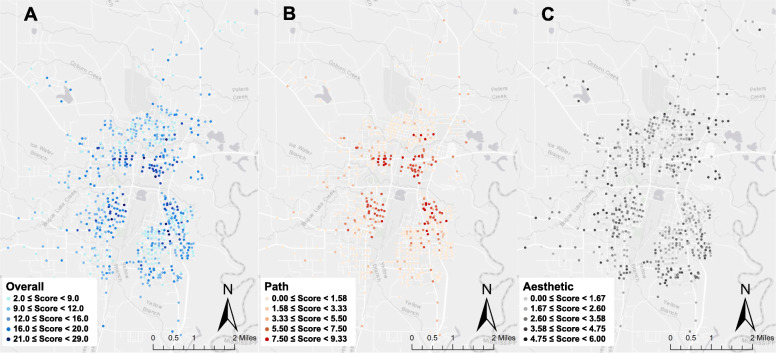
Map of average built environment scores developed for street segments in Bogalusa, Louisiana. Each colored dot indicates the mean score for the overall assessment tool (Panel **a**), the path features (Panel **b**) and the aesthetic features (Panel **c**) with darker colors indicating a higher score. Source: Authors created this map with ArcGIS 10.3 (Environmental Systems Research Institute, Redlands, CA, USA)

Street segment scores indicated the number of additional features a street segment had relative to the sample mean. Most street segments in this study had stop signs (58.7%) and public lighting (68.4%), while few had sidewalks (21.8%), any type of path (24.0%), any pedestrian signage (2.3%), or commercial or civic destinations (12.5 and 9.6%, respectively) (Supplemental Table 1). The mean *overall* score for street segments in this sample was 10.57, indicating that on average segments had 10.57 features that were more conducive to PA than the average value of that feature in the sample (Table 2). The *overall* score decreased with increasing buffer radii. The mean street segment *path score* was 2.56, which decreased to 2.25–2.27 for all buffer radii. The mean street segment *aesthetic score* was 2.82 and increased to 3.00–3.05 for all buffer radii. *Pedestrian safety*, *physical security*, *destinations* and *land use scores* did not vary substantially with changes to the radius of the buffer. Unbuffered average *overall*, *path* and *aesthetic scores* across all audits are shown for street segments in Bogalusa in Fig. 1 (panels a, b and c, respectively).

**Table 2:** Built environment scores based on the RALA* street segment audits by areal unit

	Buffer radius around participant address				
	0.00 miles	0.25 miles	0.50 miles	1.00 miles	1.50 miles
Score	Mean ± SD	Mean ± SD	Mean ± SD	Mean ± SD	Mean ± SD
Overall	10.57 ± 4.25	10.29 ± 3.69	10.25 ± 3.47	10.18 ± 3.22	10.19 ± 3.08
Path	2.56 ± 2.32	2.25 ± 2.00	2.27 ± 1.89	2.25 ± 1.71	2.25 ± 1.62
Pedestrian Safety	3.04 ± 1.78	3.02 ± 1.52	3.02 ± 1.41	3.01 ± 1.30	3.03 ± 1.21
Aesthetics	2.82 ± 1.41	3.05 ± 1.19	3.03 ± 1.11	3.00 ± 1.01	3.02 ± 0.93
Physical Security	3.71 ± 0.89	3.69 ± 0.92	3.68 ± 0.82	3.70 ± 0.73	3.72 ± 0.68
Destinations	0.55 ± 1.15	0.56 ± 0.98	0.55 ± 0.86	0.56 ± 0.75	0.56 ± 0.66
Land Use	1.72 ± 0.89	1.63 ± 0.78	1.63 ± 0.73	1.62 ± 0.66	1.60 ± 0.61

*SD* standard deviation

*RALA Rural Active Living Assessment

### Total PA

Associations between BE scores and total, transport and leisure-time PA per week are shown in Table 3. There was no association observed between the *overall score* and weekly total PA.

**Table 3:** Association between BE scores and total, transport and leisure-time physical activity (MET-minutes per week), partially (age and sex) and fully adjusted (age, sex, tract percent poverty and tract population density)

Score	Radius of Buffer around Street Segment of Residence									
	0.00 mile		0.25 mile		0.50 mile		1.00 mile		1.50 mile	
	Β (SE)	p	Β (SE)	p	Β (SE)	p	Β (SE)	p	Β (SE)	p
Partially Adjusted Model										
Total PA										
Overall	3.28 (5.21)	0.53	5.33 (6.01)	0.38	7.37 (6.38)	0.25	10.80 (6.89)	0.12	11.54 (7.21)	0.11
Path	2.36 (9.53)	0.80	10.25 (11.07)	0.35	13.49 (11.70)	0.25	19.51 (12.93)	0.13	21.69 (13.62)	0.11
Aesthetic	20.95 (15.71)	0.18	20.22 (18.63)	0.28	21.99 (20.04)	0.27	35.22 (22.07)	0.11	**43.37 (23.88)**	**0.07**
Land Use	−15.56 (24.80)	0.53	−27.33 (28.19)	0.33	−28.07 (30.46)	0.36	−38.29 (33.44)	0.25	−44.90 (36.42)	0.22
Transport PA										
Overall	−0.04 (1.08)	0.97	1.04 (1.24)	0.40	1.53 (1.32)	0.25	1.59 (1.43)	0.27	1.14 (1.50)	0.45
Path	2.42 (1.97)	0.22	**4.06 (2.29)**	**0.08**	**4.56 (2.42)**	**0.06**	4.16 (2.68)	0.12	3.09 (2.82)	0.28
Aesthetic	−1.49 (3.26)	0.65	−2.03 (3.86)	0.60	−0.65 (4.16)	0.88	0.73 (4.58)	0.87	0.96 (4.96)	0.85
Land Use	−3.52 (5.14)	0.49	−4.13 (5.84)	0.48	−3.84 (6.31)	0.54	−3.91 (6.93)	0.57	−6.67 (7.55)	0.38
Leisure-time PA										
Overall	4.19 (2.91)	0.15	**5.76 (3.35)**	**0.09**	**6.82 (3.56)**	**0.06**	**8.70 (3.85)**	0.02*	**9.20 (4.02)**	0.02*
Path	3.02 (5.32)	0.57	6.67 (6.19)	0.28	8.42 (6.54)	0.20	**12.61 (7.22)**	**0.08**	**14.76 (7.60)**	**0.05**
Aesthetic	**22.21 (8.76)**	0.01*	**26.67 (10.39)**	0.01*	**28.22 (11.17)**	0.01*	**34.77 (12.30)**	0.005*	**38.75 (13.31)**	0.004*
Land Use	−15.13 (13.85)	0.28	−7.47 (15.76)	0.64	−7.06 (17.02)	0.68	−15.70 (18.69)	0.40	−16.71 (20.36)	0.41
Fully Adjusted Model										
Total PA										
Overall	2.62 (5.96)	0.66	4.77 (6.05)	0.43	7.34 (7.11)	0.30	12.20 (8.19)	0.14	13.51 (8.81)	0.13
Path	0.77 (9.92)	0.94	9.80 (9.48)	0.30	14.22 (11.24)	0.21	**22.49 (13.48)**	**0.10**	**26.20 (15.11)**	**0.08**
Aesthetic	17.14 (16.99)	0.31	15.12 (16.79)	0.37	15.83 (17.95)	0.38	29.84 (20.84)	0.15	**38.91 (23.31)**	**0.10**
Land Use	−18.28 (22.45)	0.42	**−32.56 (18.55)**	**0.08**	−33.60 (20.87)	0.11	**−45.36 (25.01)**	**0.07**	**−53.28 (28.53)**	**0.06**
Transport PA										
Overall	−0.55 (0.93)	0.55	0.72 (1.40)	0.61	1.37 (1.63)	0.40	1.45 (1.86)	0.43	0.70 (1.96)	0.72
Path	1.97 (1.67)	0.24	**3.84 (2.18)**	**0.08**	4.53 (2.86)	0.11	3.97 (3.54)	0.26	2.28 (3.83)	0.55
Aesthetic	−1.47 (2.04)	0.47	−1.73 (2.50)	0.49	−0.03 (2.99)	0.99	1.99 (3.61)	0.58	2.66 (3.87)	0.49
Land Use	−2.29 (5.03)	0.65	−2.33 (6.31)	0.71	−1.78 (7.78)	0.82	−1.34 (7.70)	0.86	−3.92 (8.03)	0.63
Leisure-time PA										
Overall	4.00 (3.06)	0.19	5.55 (3.47)	0.11	**6.75 (4.10)**	**0.10**	**9.29 (4.96)**	**0.06**	**10.30 (5.17)**	0.05*
Path	2.08 (5.76)	0.72	6.06 (6.00)	0.31	8.28 (6.68)	0.22	**13.93 (8.36)**	**0.10**	**17.89 (9.34)**	**0.06**
Aesthetic	**17.22 (7.91)**	0.03*	**19.14 (10.00)**	**0.06**	18.85 (11.90)	0.11	23.25 (14.72)	0.11	25.71 (16.38)	0.12
Land Use	−19.84 (14.98)	0.19	−14.62 (13.38)	0.27	−14.85 (15.63)	0.34	−26.31 (18.50)	0.16	−28.93 (20.28)	0.15

*BE* built environment, *MET* metabolic equivalent, *PA* physical activity, *SE* standard error

### Transport PA

There was no association between the *overall score* and weekly transport PA. The *aesthetic score* around the residence was not associated with transport PA at any buffer size. The *land use score* around the residence was not associated with transport PA at any buffer size.

### Leisure-time PA

Each 1-point increase in the *overall score* was associated with significant 8.70 and 9.20 MET-minute higher weekly leisure-time PA in partially adjusted models in 1.00- and 1.50-mile buffers around the residence, and with a significant 10.30 MET-minute higher weekly leisure-time PA in the 1.50-mile buffer in fully adjusted models (all *p* < 0.05). Each 1-point increase in the *aesthetic score* was associated with a statistically significant 22.21 to 38.75 MET-minute higher weekly leisure-time PA in all areal units (all *p* < 0.05) in the partially adjusted model. In the fully adjusted model, *aesthetic score* was associated with a significant 17.22 MET-minute higher weekly leisure-time PA for the street segment of residence (*p* < 0.05). There was no association between the *land use score* and leisure-time PA.

Results of GEE models for the categories of features with no associations with PA are presented in Supplemental Table 4. *Physical security* demonstrated significant associations with leisure-time PA in 1.00- and 1.50-mile buffers in partially and fully adjusted models, but due to the low reliability of the *physical security score* these results are not presented in the main results and will not be discussed further.

### Non-statistically significant trends

#### Total PA

The street segment *path score* was not associated with total PA in partially adjusted models, but a 1-point increase in *path score* was associated with 22.49 and 26.20 MET-minutes higher weekly total PA in 1.00- and 1.50-mile buffers around the residence (0.05 < *p* < 0.1). Each 1-point increase in the *aesthetic score* at the 1.50-mile buffer was associated with 43.4 and 38.9 MET-minutes higher weekly total PA in partially and fully adjusted models, respectively (0.05 < *p* < 0.1). Each 1-point increase in the *land use* score in the fully adjusted model was associated with 32.6, 45.4 and 53.3 MET-minutes lower weekly total PA in 0.25-, 1.00- and 1.50-mile buffers, respectively (0.05 < *p* < 0.1).

#### Transport PA

Each 1-point increase in the *path score* was associated with 4.06 and 4.56 MET-minutes higher weekly transport PA in 0.25- and 0.50-mile buffers in the partially adjusted model (0.05 < *p* < 0.1), and 3.84 MET-minutes higher weekly transport PA in the 0.25-mile buffer in the fully adjusted model (0.05 < *p* < 0.1).

#### Leisure-time PA

A 1-point increase in the *path score* at 1.00 and 1.50-mile buffers with was associated with 12.61 and 14.76 MET-minutes higher weekly leisure-time PA in the partially adjusted model, and 13.93 and 17.89 MET-minutes higher weekly leisure-time PA in the fully adjusted model. In the fully adjusted model, aesthetic score was associated with 19.14 MET-minutes higher leisure-time PA in the 0.25-mile buffer (0.05 < *p* < 0.1).

## Discussion

Reported weekly PA was low among participants in the Bogalusa Heart Study, and few street segments had features supportive of PA such as paths (24.0%) or pedestrian signage (2.3%). Significant associations were observed between scores summarizing BE features around the residence and PA for participants in the Bogalusa Heart Study. In partially adjusted models, significant associations were identified between the *overall score* and higher leisure-time PA in buffers of 1.00- and 1.50-miles around the residence and between the *aesthetic score* and higher leisure-time PA in all buffers. In fully adjusted models, significant associations between the *overall score* and higher leisure-time PA remained for the 1.50-mile buffer, and between the *aesthetic score* and higher leisure-time PA for the street segment of residence. No significant associations were observed between BE scores around the home and total PA or transport PA, though there were suggestive associations between the *path score* and transport PA in 0.25- and 0.50-mile and between *path*, *aesthetic* and *land use scores* and total PA in 1.00- and 1.50-mile buffers.

Streets segments in this study were similar to those in other studies of street characteristics in the rural South, with inadequate pedestrian safety features and few destinations [29]. Previous research on the relationship between BE features and PA in rural populations has found relationships between pleasant aesthetics, the presence of trails, parks and walkable destinations and safety and higher levels of PA [15]. A recently published study found that poor cardiovascular health behaviors among Maine residents responding to the Behavioral Risk Factor Surveillance System survey between 2011 and 2014, including low levels of PA, were more likely among individuals living in areas with low fitness facility density at a county level [30]. While the present study did not create an index of fitness facility density, the *overall score* represents an index of the relative density of features that support PA on the segment of residence, and this was associated with leisure-time PA in a 1.50-mile buffer. Similar to the association between the *aesthetic scores* and PA identified in this study, a prior contextual analysis of nonmetropolitan areas identified associations between natural amenities and increased PA [31]. Non-significant associations between the *path score* and higher total, transport and leisure-time PA were identified in this study, similar to prior to studies that have reported significant associations between perceived or objective path features and increased PA [31, 32].

The lack of a significant association between the *path score* and PA, especially transport PA, was unexpected. Prior research has identified associations between perceived environmental features and regular walking [33] and utilitarian walking in a small town settings [34]. These perceived environmental features associated with increased odds of utilitarian walking included crosswalks and crossing signals, slower traffic speeds, the presence of a path and the presence of a natural recreation area in the neighborhood of residence [34]. Participants in that study reported high levels of utilitarian walking, with 73% reporting any utilitarian walking and 22% reporting 150 or more minutes of utilitarian walking per week [34]. The participants in the present study engaged in much less transport PA, with an average of 43.5 MET-minutes of transport PA per week (equivalent to 13.2 min of transport walking per week). Even at such low levels of transport PA, there was a suggestive (though not statistically significant) association between the path score and transport PA, which may be associated more strongly with transport PA in a population that engages in more regular walking. Over a third of adults in the United States reported being regular walkers, getting at least 150 min of moderate-to-vigorous PA per week [32], which is far above the amount of transport PA observed in this study, so further exploration of the relationship between path features and transport PA in rural adults is merited.

The suggestive (though not statistically significant) associations between higher *land-use scores* and lower total PA in fully adjusted models was also unexpected. A higher land-use score indicates land development types thought be associated with higher PA, such as the presence of mixed development, the absence of terrain that would dissuade PA such as hills, and more dense residential settlement with homes in better condition. Most segments had residential development only (86.6%) and flat terrain (94.9%). The inverse association between our *land-use score* and PA conflicts with a prior reported association found between the objectively (geographic information systems) measured presence of land with an industrial use, which was associated with any and higher levels of PA in a prior analysis of rural BE and PA [34]. Another study demonstrated relationships between environmental characteristics (sunlight hours, mean temperature, percent humidity, topographic relief) and increased PA in a study using broader geographic units of exposure [31]. The majority of participants in this study live in southern Louisiana and adjacent areas of southern Mississippi, with very little variability in climate and relatively consistent topography, which when coupled with the more fine geographic units used could explain the deviation from previously published results.

Previous research has suggested that the relationship between BE features and PA is likely moderated by individual perception of the environment [35]. Perceptions of the environment have been associated with PA in rural-dwelling adults [34] and this increase in PA with perceptions of more PA-supportive features and facilities may be due to the removal of a psychological barrier to PA.

This study has a number of strengths that should be mentioned. A validated instrument was used to assess PA among study participants [23], and this also allowed for the evaluation of different domains of PA. A street segment audit instrument developed for rural environment assessment was used to conduct BE audits [19]. We have provided detailed information on our scoring procedure that can be replicated where one previously did not exist for the instrument. This responds to a call from The Community Guide to refine summary assessment tools for objective characteristics of the environment [36]. Objective assessment of BE along the street segment of residence of each participant was conducted for every available image in Google StreetView. Duplicate audits conducted on a randomly selected subset of segments to evaluate reliability, which was found to be good. The high density of street segments audited in Bogalusa allowed for the calculation of unique “neighborhood” scores for different size buffers around each segment of residence. This allowed the evaluation of BE influences across different size geographic units, because there is little knowledge about the appropriate areal unit for rural BE exposures. There are also limitations which should be mentioned. The associations observed are cross sectional, and future research into the longitudinal relationship between BE and PA in rural settings is necessary. The use of Google Street View is a limitation as the street segment images may not have been from the same time as the reports of PA behavior. Although the segment images used were the one closest in time to the study visit, there may be changes to the environment that were not captured. The appropriate geographic context in which BE influences PA for these study participants is unknown [37]. This study used geographic contexts around the residence as the unit of study, which could lead to non-differential misclassification of BE exposures and underestimation of the influence of the BE on PA. The participants in this study are primarily white and black middle-aged adults in rural Louisiana, and reported associations might not be generalizable to other age groups and races, or to other geographies.

## Conclusions

Because rural areas will face resource constraints, feasibility should be considered when evaluating BE modifications to promote PA [17]. Prior studies have identified feasible changes that can be made to promote PA, including enhancing existing features, and making small improvements [17]. The significant association between the aesthetic score and increased leisure-time PA indicates that small interventions to improve community aesthetics (the removal of incivilities such as abandoned vehicles, removal of garbage and litter, encouraging the proper disposal of tobacco products, and installing and maintaining greenspaces) can contribute to more active rural communities.

## Supplementary information

**Additional file 1: Supplemental Fig. 1.** Graphic demonstrating the use of buffers in the construction of buffered neighborhood scores. The hexagon markings indicate sample audited street segments with buffers of 0.25, 0.50 and 1.00 miles around example segments A and B.

**Additional file 2: Supplemental Table 1.** Agreement for specific items assessed using a modified Rural Active Living Assessment street segment audit tool on a sample of street segments of residence for participants in the Bogalusa Heart Study and description of the development of overall and category specific scores for street segment built environment. Comm: commercial, Dest: destination, Freq: frequency, Mod: moderate, mph: miles per hour, Res: residential.

**Additional file 3: Supplemental Table 2.** Agreement for all items overall and in categories of features assessed using a modified Rural Active Living Assessment street segment audit tool on a sample of street segments of residence for participants in the Bogalusa Heart Study (*n* = 196 segments, 392 observations).

**Additional file 4: Supplemental Table 3.** Intra-class correlation coefficients for scores developed overall and in categories of features assessed using a modified Rural Active Living Assessment street segment audit tool on a sample of street segments of residence for participants in the Bogalusa Heart Study (n = 196 segments, 392 observations). *The audited street segment image year was classified as during or before 2010 or after 2010.

**Additional file 5: Supplemental Table 4.** Association between BE scores and total, transport and leisure-time physical activity (MET-minutes per week), partially (age and sex) and fully adjusted (age, sex, tract percent poverty and tract population density). BE: built environment; SE: standard error; PA: physical activity.

## Data Availability

The data are available upon reasonable request to the BHS Steering Committee. A portion of the data are available through the NHLBI BioLINCC repository.

## Abbreviations

BE: build environment
BMI: body mass index
FPL: federal poverty level
GEE: generalized estimating equations
IPAQ: International Physical Activity Questionnaire
MET: metabolic equivalent
PA: physical activity
RALA: Rural Active Living Assessment
