# Supplementary material for: Cross-sectional associations between the neighborhood built environment and physical activity in a rural setting: the Bogalusa Heart Study
Source: BMC Public Health. 2020 Sep 18;20:1426. doi: 10.1186/s12889-020-09509-4 (PMC7501650; doi:10.1186/s12889-020-09509-4)
Supplement: Supplementary file 1 — Additional file 1: Supplemental Fig. 1. Graphic demonstrating the use of buffers in the construction of buffered neighborhood scores. The hexagon markings indicate sample audited street segments with buffers of 0.25, 0.50 and 1.00 miles around example segments A and B. [file 12889_2020_9509_MOESM1_ESM.pdf]

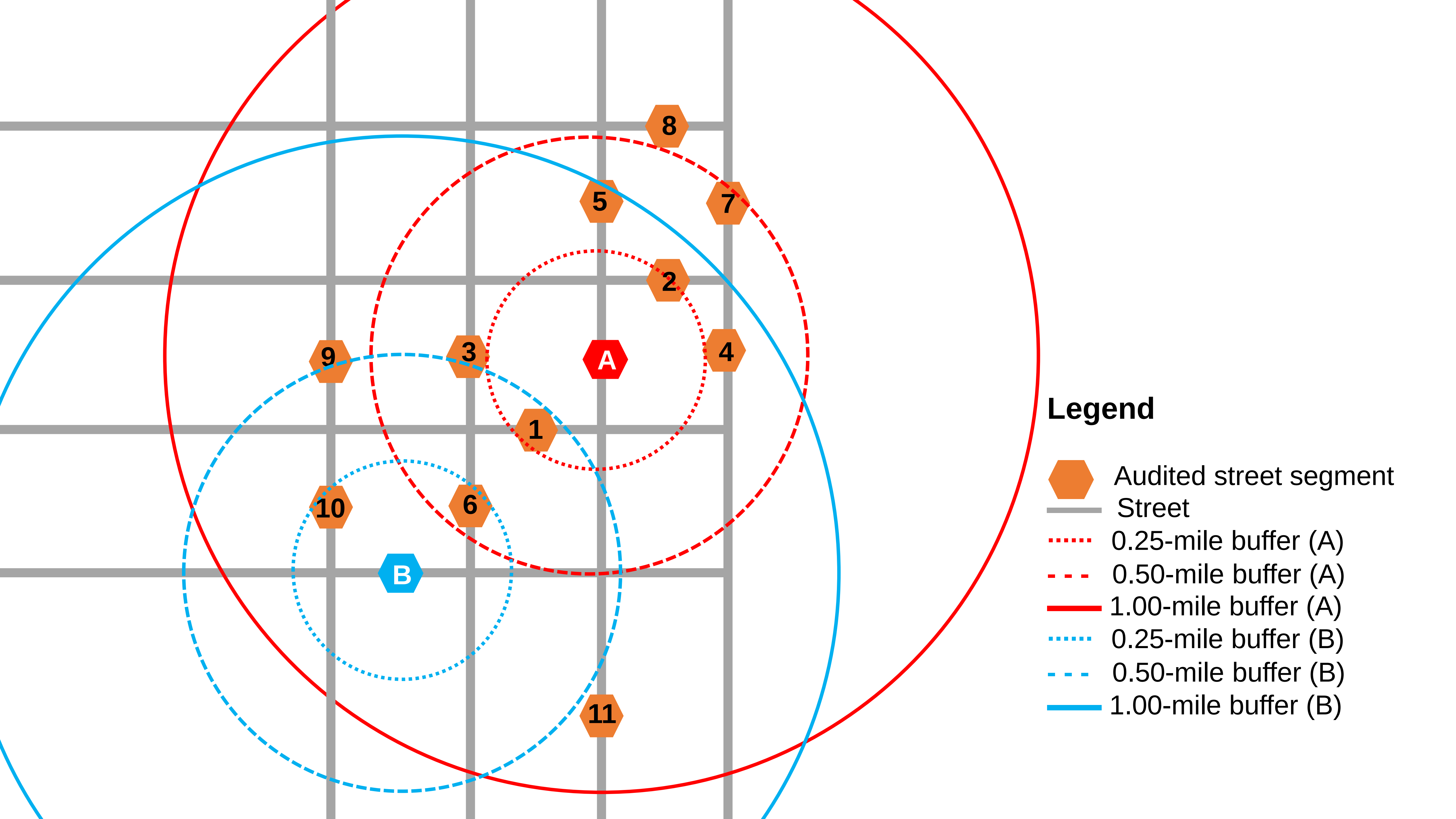
8
5
7
2
9
3
A
4
1
10
6
B
11
Legend
Audited street segment
Street
0.25-mile buffer (A)
0.50-mile buffer (A)
1.00-mile buffer (A)
0.25-mile buffer (B)
0.50-mile buffer (B)
1.00-mile buffer (B)
