# Supplementary material for: Cross-sectional associations between the neighborhood built environment and physical activity in a rural setting: the Bogalusa Heart Study
Source: BMC Public Health. 2020 Sep 18;20:1426. doi: 10.1186/s12889-020-09509-4 (PMC7501650; doi:10.1186/s12889-020-09509-4)
Supplement: Supplementary file 2 — Additional file 2: Supplemental Table 1. Agreement for specific items assessed using a modified Rural Active Living Assessment street segment audit tool on a sample of street segments of residence for participants in the Bogalusa Heart Study and description of the development of overall and category specific scores for street segment built environment. Comm: commercial, Dest: destination, Freq: frequency, Mod: moderate, mph: miles per hour, Res: residential. [file 12889_2020_9509_MOESM2_ESM.docx]

**Supplemental Table 1.** Agreement for specific items assessed using a modified Rural Active Living Assessment street segment audit tool on a sample of street segments of residence for participants in the Bogalusa Heart Study and description of the development of overall and category specific scores for street segment built environment.

|  | | Percent | |  | Score Development | | | | | | |
| --- | --- | --- | --- | --- | --- | --- | --- | --- | --- | --- | --- |
|  | |  |  |  | N=2648 | | | | | | |
| Category | | N=2648 | N=196 |  | Overall Score | | |  | Category Specific Score | | |
|  | Variable | Freq. | Agree |  | Feature Present (mean) | Feature Absent (mean) | Keep |  | Feature Present (mean) | Feature Absent (mean) | Keep |
| **Path features** | |  |  |  |  |  |  |  |  |  |  |
|  | Sidewalk present | 21.8 | 89.5 |  | 26.7 | 19.5 | Yes |  | 8.0 | 2.5 | Yes |
|  | Any path present | 24.0 | 94.6 |  | 26.3 | 19.4 | Yes |  | 7.7 | 2.4 | Yes |
|  | Path is attractive | 10.8 | 87.7 |  | 26.3 | 19.4 | Yes |  | 7.7 | 2.4 | Yes |
|  | Path is wide enough | 14.7 | 88.0 |  | 19.4 | 25.4 | **No** |  | 2.4 | 6.9 | **No** |
|  | Connector is present | 22.5 | 68.1 |  | 25.9 | 19.6 | Yes |  | 7.5 | 2.5 | Yes |
|  | Connector in good condition | 9.4 | 66.9 |  | 25.9 | 19.6 | Yes |  | 7.5 | 2.5 | Yes |
|  | Segment is walkable | 29.8 | 27.7 |  | 24.6 | 19.5 | Yes |  | 6.4 | 2.5 | Yes |
|  | Shoulder in good condition | 9.3 | 88.6 |  | 26.5 | 19.7 | Yes |  | 8.0 | 2.6 | Yes |
|  | Buffer in good condition | 3.1 | 91.0 |  | 22.9 | 20.9 | Yes |  | 5.5 | 3.5 | Yes |
|  | Buffer/shoulder presence | 27.0 | 88.9 |  | 25.2 | 19.5 | Yes |  | 7.1 | 2.4 | Yes |
|  | No barriers present | 91.9 | 88.0 |  | 21.2 | 19.6 | Yes |  | 3.7 | 2.7 | **No** |
| **Pedestrian safety features** | |  |  |  |  |  |  |  |  |  |  |
|  | Crosswalk present | 3.8 | 98.5 |  | 28.9 | 20.7 | Yes |  | 7.2 | 4.4 | Yes |
|  | Crossing signal present | 0.8 | 99.1 |  | 30.7 | 21.0 | Yes |  | 8.1 | 4.5 | Yes |
|  | Pedestrian signage present | 2.3 | 98.5 |  | 27.2 | 20.9 | Yes |  | 7.3 | 4.4 | Yes |
|  | Children at play sign present | 13.1 | 86.1 |  | 22.9 | 20.8 | Yes |  | 6.7 | 4.1 | Yes |
|  | Any signage present | 18.3 | 86.1 |  | 24.3 | 20.3 | Yes |  | 6.8 | 4.0 | Yes |
|  | Traffic light present | 3.6 | 98.2 |  | 27.3 | 20.8 | Yes |  | 6.1 | 4.4 | Yes |
|  | Stop sign present | 58.7 | 77.7 |  | 21.5 | 20.3 | Yes |  | 5.1 | 3.6 | Yes |
|  | Speed bump present | 0.4 | 99.7 |  | 26.7 | 21.0 | Yes |  | 7.7 | 4.5 | Yes |
|  | Public lighting present | 68.4 | 65.1 |  | 22.3 | 18.4 | Yes |  | 5.2 | 3.0 | Yes |
|  | Any safety features present | 80.7 | 81.9 |  | 21.8 | 17.9 | Yes |  | 5.0 | 2.3 | Yes |
|  | Paved road | 98.7 | 77.7 |  | 21.2 | 20.9 | **No** |  | 4.5 | 4.5 | **No** |
|  | Road in good condition | 54.1 | 49.1 |  | 22.4 | 19.5 | Yes |  | 5.0 | 3.9 | Yes |
|  | Speed limit posted | 24.4 | 90.1 |  | 23.3 | 20.3 | Yes |  | 5.9 | 4.0 | Yes |
|  | Speed limit, mean mph | 29.9 | 85.3 |  | 24.4 | 20.6 | Yes |  | 6.9 | 4.1 | Yes |
|  | Low traffic volume | 69.4 | 76.5 |  | 20.8 | 21.6 | **No** |  | 4.7 | 4.1 | **No** |
| **Segment Aesthetics** | |  |  |  |  |  |  |  |  |  |  |
|  | No incivility present | 66.2 | 73.8 |  | 22.2 | 18.8 | Yes |  | 5.6 | 4.3 | Yes |
|  | No tobacco litter present | 68.9 | 77.4 |  | 21.3 | 20.5 | **No** |  | 5.6 | 3.3 | Yes |
|  | Light or no garbage/litter | 70.1 | 24.4 |  | 22.1 | 18.5 | Yes |  | 5.6 | 3.5 | Yes |
|  | Greenspace present | 99.5 | 99.4 |  | 21.0 | 23.7 | **No** |  | 5.9 | 4.8 | Yes |
|  | >50%greenspace maintained | 72.4 | 21.7 |  | 23.4 | 19.6 | Yes |  | 5.6 | 4.4 | Yes |
|  | Aesthetically pleasing | 30.0 | 50.0 |  | 23.6 | 20.0 | Yes |  | 5.7 | 4.8 | **No** |
| **Land Use** | |  |  |  |  |  |  |  |  |  |  |
|  | Industry/agriculture | 4.8 | 90.7 |  | 21.2 | 21.0 | **No** |  | 4.6 | 2.1 | Yes |
|  | Light industry | 1.2 | 97.0 |  | 20.1 | 21.0 | **No** |  | 4.2 | 2.6 | Yes |
|  | Heavy industry | 0.1 | 99.4 |  | 18.0 | 21.0 | **No** |  | 4.5 | 1.9 | Yes |
|  | Farmland | 3.4 | 94.3 |  | 21.5 | 21.0 | **No** |  | 3.7 | 2.6 | Yes |
|  | Other industry/agriculture | 0.3 | 99.4 |  | 21.6 | 21.0 | **No** |  | 4.9 | 3.0 | Yes |
|  | Residential only land use | 86.6 | 71.1 |  | 22.4 | 20.8 | Yes |  | 5.1 | 3.1 | Yes |
|  | Flat terrain | 94.9 | 89.2 |  | 20.4 | 21.1 | **No** |  | 4.9 | 2.9 | Yes |
|  | ≥Mod. residential density | 75.4 | 60.8 |  | 21.5 | 19.5 | Yes |  | 4.7 | 2.9 | Yes |
|  | Single family home present | 92.3 | 55.1 |  | 21.9 | 19.7 | Yes |  | 3.0 | 3.0 | **No** |
|  | Multi-family home present | 8.2 | 93.1 |  | 25.2 | 20.7 | Yes |  | 5.0 | 2.9 | Yes |
|  | Mobile home present | 19.2 | 80.1 |  | 20.1 | 21.3 | **No** |  | 5.3 | 3.0 | Yes |
|  | Other dwelling present | 7.5 | 88.5 |  | 21.5 | 21.0 | **No** |  | 3.2 | 2.9 | **No** |
|  | Any dwelling present | 96.0 | 94.6 |  | 21.1 | 20.4 | **No** |  | 3.9 | 2.9 | **No** |
| **Physical Security** | |  |  |  |  |  |  |  |  |  |  |
|  | No fencing, comm. | 98.8 | 100.0 |  | 21.0 | 21.3 | **No** |  | 3.1 | 2.7 | **No** |
|  | No security blinds, comm. | 99.2 | 99.1 |  | 21.0 | 22.0 | **No** |  | 3.3 | 2.5 | **No** |
|  | No window bars, comm. | 98.9 | 99.1 |  | 21.0 | 23.1 | **No** |  | 3.7 | 2.9 | **No** |
|  | No window bars, res. | 77.1 | 22.9 |  | 21.1 | 21.0 | **No** |  | 3.8 | 2.8 | Yes |
|  | No murals | 99.5 | 99.7 |  | 21.0 | 22.3 | **No** |  | 4.2 | 2.9 | Yes |
|  | No security signage | 87.7 | 85.8 |  | 21.0 | 21.2 | **No** |  | 3.0 | 1.3 | Yes |
| **Destinations** | |  |  |  |  |  |  |  |  |  |  |
|  | Any public destination | 9.6 | 91.9 |  | 23.7 | 20.8 | Yes |  | 2.7 | 0.4 | Yes |
|  | Library present | 0.1 | 100.0 |  | 25.0 | 21.0 | Yes |  | 6.0 | 0.6 | Yes |
|  | Museum present | 0.0 | 100.0 |  | - | 21.0 | **No** |  | - | 0.6 | **No** |
|  | Community center present | 0.2 | 100.0 |  | 27.8 | 21.0 | Yes |  | 4.0 | 0.6 | Yes |
|  | Post office present | 0.2 | 100.0 |  | 30.4 | 21.0 | Yes |  | 4.0 | 0.6 | Yes |
|  | Town office present | 0.2 | 100.0 |  | 25.8 | 21.0 | Yes |  | 3.6 | 0.6 | Yes |
|  | Courthouse present | 0.1 | 99.7 |  | 28.0 | 21.0 | Yes |  | 5.0 | 0.6 | Yes |
|  | Fire station present | 0.5 | 99.7 |  | 23.0 | 21.0 | Yes |  | 2.7 | 0.6 | Yes |
|  | Church present | 6.3 | 93.1 |  | 23.0 | 20.9 | Yes |  | 2.5 | 0.5 | Yes |
|  | Hospital present | 0.4 | 99.7 |  | 24.0 | 21.0 | Yes |  | 3.8 | 0.6 | Yes |
|  | Athletic facility present | 0.5 | 99.4 |  | 23.0 | 21.0 | Yes |  | 2.4 | 0.6 | Yes |
|  | Playground present | 0.9 | 99.1 |  | 29.2 | 21.0 | Yes |  | 3.4 | 0.6 | Yes |
|  | Other civic facility present | 1.2 | 97.3 |  | 24.0 | 21.0 | Yes |  | 2.9 | 0.6 | Yes |
|  | Restaurant present | 3.0 | 97.3 |  | 26.6 | 20.9 | Yes |  | 4.0 | 0.5 | Yes |
|  | Bar present | 0.3 | 100.0 |  | 23.0 | 21.0 | Yes |  | 4.3 | 0.6 | Yes |
|  | Fast food present | 1.1 | 99.1 |  | 27.6 | 21.0 | Yes |  | 4.7 | 0.6 | Yes |
|  | Food market present | 0.9 | 99.1 |  | 24.7 | 21.0 | Yes |  | 3.6 | 0.6 | Yes |
|  | Theater present | 0.1 | 100.0 |  | 27.0 | 21.0 | Yes |  | 7.5 | 0.6 | Yes |
|  | Gas station present | 2.0 | 98.2 |  | 26.9 | 20.9 | Yes |  | 4.2 | 0.5 | Yes |
|  | Convenience store present | 3.4 | 98.5 |  | 27.0 | 20.9 | Yes |  | 4.5 | 0.5 | Yes |
|  | Small retail present | 1.1 | 99.1 |  | 26.5 | 20.8 | Yes |  | 4.5 | 0.5 | Yes |
|  | Big box retail present | 0.1 | 99.1 |  | 26.2 | 21.0 | Yes |  | 4.6 | 0.6 | Yes |
|  | Fitness center present | 0.0 | 100.0 |  | 27.7 | 21.0 | Yes |  | 4.3 | 0.6 | Yes |
|  | Private medical present | 1.0 | 99.4 |  | 24.9 | 21.0 | Yes |  | 3.9 | 0.6 | Yes |
|  | Private other present | 2.2 | 95.8 |  | 26.6 | 20.9 | Yes |  | 4.0 | 0.5 | Yes |
|  | Other commercial dest. | 7.5 | 95.8 |  | 24.2 | 20.8 | Yes |  | 3.4 | 0.4 | Yes |
|  | Any commercial dest. | 12.5 | 91.9 |  | 24.4 | 20.6 | Yes |  | 3.3 | 0.2 | Yes |
|  | Any school present | 2.2 | 98.8 |  | 26.3 | 20.9 | Yes |  | 3.2 | 0.6 | Yes |
|  | Elementary school present | 0.9 | 98.8 |  | 26.4 | 21.0 | Yes |  | 3.3 | 0.6 | Yes |
|  | Middle school present | 0.3 | 99.7 |  | 24.9 | 21.0 | Yes |  | 2.0 | 0.6 | Yes |
|  | High school present | 0.4 | 100.0 |  | 25.9 | 21.0 | Yes |  | 2.9 | 0.6 | Yes |
|  | Private school present | 0.1 | 100.0 |  | 29.0 | 21.0 | Yes |  | 5.0 | 0.6 | Yes |
|  | Other school present | 0.5 | 99.4 |  | 27.5 | 21.0 | Yes |  | 4.1 | 0.6 | Yes |

Comm: commercial, Dest: destination, Freq: frequency, Mod: moderate, mph: miles per hour, Res: residential
