# Supplementary material for: Cross-sectional associations between the neighborhood built environment and physical activity in a rural setting: the Bogalusa Heart Study
Source: BMC Public Health. 2020 Sep 18;20:1426. doi: 10.1186/s12889-020-09509-4 (PMC7501650; doi:10.1186/s12889-020-09509-4)
Supplement: Supplementary file 3 — Additional file 3: Supplemental Table 2. Agreement for all items overall and in categories of features assessed using a modified Rural Active Living Assessment street segment audit tool on a sample of street segments of residence for participants in the Bogalusa Heart Study (n = 196 segments, 392 observations). [file 12889_2020_9509_MOESM3_ESM.docx]

**Supplemental Table 2**. Agreement for all items overall and in categories of features assessed using a modified Rural Active Living Assessment street segment audit tool on a sample of street segments of residence for participants in the Bogalusa Heart Study (n=196 segments, 392 observations).

| Category |  | Simple Kappa | Weighted Kappa | % Agreement |
| --- | --- | --- | --- | --- |
| Overall (all categories) |  | 0.76 | 0.90 | 87.68 |
| Path Features |  | 0.63 | 0.93 | 79.87 |
| Pedestrian Safety Features |  | 0.75 | 0.89 | 84.63 |
| Segment Aesthetics |  | 0.25 | 0.32 | 57.78 |
| Land Use |  | 0.74 | 0.65 | 85.63 |
| Physical Security |  | 0.77 | 0.74 | 84.44 |
| Destinations |  | 0.34 | 0.37 | 98.47 |
