# Supplementary material for: Cross-sectional associations between the neighborhood built environment and physical activity in a rural setting: the Bogalusa Heart Study
Source: BMC Public Health. 2020 Sep 18;20:1426. doi: 10.1186/s12889-020-09509-4 (PMC7501650; doi:10.1186/s12889-020-09509-4)
Supplement: Supplementary file 4 — Additional file 4: Supplemental Table 3. Intra-class correlation coefficients for scores developed overall and in categories of features assessed using a modified Rural Active Living Assessment street segment audit tool on a sample of street segments of residence for participants in the Bogalusa Heart Study (n = 196 segments, 392 observations). *The audited street segment image year was classified as during or before 2010 or after 2010. [file 12889_2020_9509_MOESM4_ESM.docx]

**Supplemental Table 3**. Intra-class correlation coefficients for scores developed overall and in categories of features assessed using a modified Rural Active Living Assessment street segment audit tool on a sample of street segments of residence for participants in the Bogalusa Heart Study (n=196 segments, 392 observations).

| Category |  | Uniform Scoring | Stratified by Auditor | Stratified by Auditor, Year* |
| --- | --- | --- | --- | --- |
| Overall (all categories) |  | 0.60 | 0.62 | 0.59 |
| Path Features |  | 0.64 | 0.74 | 0.73 |
| Pedestrian Safety Features |  | 0.53 | 0.55 | 0.54 |
| Segment Aesthetics |  | 0.14 | 0.34 | 0.35 |
| Land Use |  | 0.29 | 0.08 | 0.20 |
| Physical Security |  | 0.00 | 0.04 | 0.03 |
| Destinations |  | 0.47 | 0.46 | 0.49 |

*The audited street segment image year was classified as during or before 2010 or after 2010.
