# Supplementary material for: Cross-sectional associations between the neighborhood built environment and physical activity in a rural setting: the Bogalusa Heart Study
Source: BMC Public Health. 2020 Sep 18;20:1426. doi: 10.1186/s12889-020-09509-4 (PMC7501650; doi:10.1186/s12889-020-09509-4)
Supplement: Supplementary file 5 — Additional file 5: Supplemental Table 4. Association between BE scores and total, transport and leisure-time physical activity (MET-minutes per week), partially (age and sex) and fully adjusted (age, sex, tract percent poverty and tract population density). BE: built environment; SE: standard error; PA: physical activity. [file 12889_2020_9509_MOESM5_ESM.docx]

**Supplemental Table 4.** Association between BE scores and total, transport and leisure-time physical activity (MET-minutes per week), partially (age and sex) and fully adjusted (age, sex, tract percent poverty and tract population density).

|  |  | Buffer Around the Residence | | | | | | | | | |
| --- | --- | --- | --- | --- | --- | --- | --- | --- | --- | --- | --- |
|  |  | 0.0 mile | | 0.25 mile | | 0.50 mile | | 1.00 mile | | 1.50 mile | |
|  | Score | Β (SE) | p | Β (SE) | p | Β (SE) | p | Β (SE) | p | Β (SE) | p |
|  |  | Partially Adjusted Model | | | | | | | | | |
| Total PA | |  |  |  |  |  |  |  |  |  |  |
|  | Pedestrian safety | -0.22 (12.46) | 0.99 | 6.53 (14.60) | 0.66 | 10.52 (15.68) | 0.50 | 17.29 (17.03) | 0.31 | 19.47 (18.36) | 0.29 |
|  | Physical Security | -23.65 (24.90) | 0.34 | 5.04 (23.98) | 0.83 | 20.62 (26.86) | 0.44 | 38.24 (30.17) | 0.21 | 52.67 (32.62) | 0.11 |
|  | Destination | 17.22 (19.18) | 0.37 | 21.51 (22.49) | 0.34 | 23.87 (25.63) | 0.35 | 19.26 (29.26) | 0.51 | 8.94 (33.35) | 0.79 |
| Transport PA | |  |  |  |  |  |  |  |  |  |  |
|  | Pedestrian safety | 0.13 (2.58) | 0.96 | 1.98 (3.03) | 0.51 | 2.22 (3.25) | 0.49 | 3.65 (3.53) | 0.30 | 3.80 (3.81) | 0.32 |
|  | Physical Security | -2.20 (5.16) | 0.67 | -0.98 (4.97) | 0.84 | 2.68 (5.57) | 0.63 | 1.31 (6.26) | 0.83 | -0.15 (6.77) | 0.98 |
|  | Destination | -3.54 (3.98) | 0.37 | -0.90 (4.66) | 0.85 | -0.73 (5.31) | 0.89 | -2.54 (6.07) | 0.68 | -5.45 (6.91) | 0.43 |
| Leisure-time PA | |  |  |  |  |  |  |  |  |  |  |
|  | Pedestrian safety | 2.19 (6.96) | 0.75 | 4.97 (8.16) | 0.54 | 7.52 (8.76) | 0.39 | 9.70 (9.51) | 0.31 | 9.45 (10.26) | 0.36 |
|  | Physical Security | -3.05 (13.91) | 0.83 | 14.92 (13.39) | 0.27 | 22.75 (15.00) | 0.13 | **37.64 (16.84)** | **0.03*** | **47.11 (18.20)** | **0.01*** |
|  | Destination | 12.99 (10.71) | 0.23 | 14.11 (12.57) | 0.26 | 11.74 (14.32) | 0.41 | 6.98 (16.35) | 0.67 | 3.53 (18.63) | 0.85 |
|  | | Fully Adjusted Model | | | | | | | | | |
| Total PA | |  |  |  |  |  |  |  |  |  |  |
|  | Pedestrian safety | 0.46 (12.51) | 0.97 | 7.24 (13.31) | 0.59 | 11.60 (14.77) | 0.43 | 19.39 (16.93) | 0.25 | 22.12 (19.46) | 0.26 |
|  | Physical Security | -22.19 (25.17) | 0.38 | 6.36 (21.72) | 0.77 | 22.27 (24.67) | 0.37 | 39.87 (31.02) | 0.20 | 54.15 (36.72) | 0.14 |
|  | Destination | 16.75 (20.48) | 0.41 | 21.26 (22.94) | 0.35 | 22.69 (25.92) | 0.38 | 17.40 (28.32) | 0.54 | 6.97 (31.74) | 0.83 |
| Transport PA | |  |  |  |  |  |  |  |  |  |  |
|  | Pedestrian safety | -1.03 (2.17) | 0.63 | 0.79 (2.47) | 0.75 | 0.92 (2.98) | 0.76 | 2.38 (3.37) | 0.48 | 2.39 (3.73) | 0.52 |
|  | Physical Security | -2.47 (3.06) | 0.42 | -1.51 (3.51) | 0.67 | 2.06 (5.10) | 0.69 | 0.51 (6.24) | 0.93 | -1.04 (6.78) | 0.88 |
|  | Destination | -3.76 (2.51) | 0.13 | -1.12 (2.80) | 0.69 | -1.22 (3.71) | 0.74 | -3.57 (4.06) | 0.38 | -7.19 (4.99) | 0.15 |
| Leisure-time PA | |  |  |  |  |  |  |  |  |  |  |
|  | Pedestrian safety | 4.74 (7.18) | 0.51 | 7.31 (8.54) | 0.39 | 10.19 (9.44) | 0.28 | 12.57 (12.20) | 0.30 | 12.57 (12.20) | 0.30 |
|  | Physical Security | -2.02 (13.63) | 0.88 | 16.03 (13.90) | 0.25 | 24.09 (16.55) | 0.15 | **48.23 (23.96)** | **0.04*** | **48.23 (23.96)** | **0.04*** |
|  | Destination | 12.49 (11.85) | 0.29 | 13.21 (13.38) | 0.32 | 10.50 (15.48) | 0.50 | 3.98 ( 19.32) | 0.84 | 3.98 (19.32) | 0.84 |

BE: built environment; SE: standard error; PA: physical activity
